# Prolactin Mediates Distinct Time Course Regulation of Tyrosine Hydroxylase Phosphorylation and Gene Expression in Tuberoinfundibular Dopaminergic Neurons of Female Rats

**DOI:** 10.3390/cells14090642

**Published:** 2025-04-27

**Authors:** Philip J. Jensik, Lydia A. Arbogast

**Affiliations:** Division of Molecular and Integrative Physiology, Department of Biomedical Science, Southern Illinois University School of Medicine, Carbondale, IL 62901-6523, USA; pjensik@siu.edu

**Keywords:** prolactin, dopamine, TIDA, prolactin receptor signaling, tyrosine hydroxylase, phosphorylation, gene expression, promoter activity, JAK2, STAT5

## Abstract

Prolactin (PRL) regulates its own secretion by short-loop feedback to tuberoinfundibular dopaminergic (TIDA) neurons. PRL-induced cellular mechanisms in the regulation of tyrosine hydroxylase (TH) are not completely understood. The objectives were to (1) examine PRL-induced, time-dependent hypothalamic changes in JAK2-STAT5B signaling, TH activity, TH phosphorylation state and *Th* mRNA levels, and (2) evaluate direct influences of PRLR-STAT5B signaling on *Th* promoter activity. Ovariectomized rats were administered ovine PRL. JAK2 and STAT5 phosphorylation in the mediobasal hypothalamus peaked at 15 and 30–60 min, respectively. TH Ser40 phosphorylation in the median eminence was increased between 2 and 72 h, correlating with increased dihydroxyphenylalanine (DOPA) accumulation. *Th* mRNA levels in TIDA neurons were unchanged up to 72 h but elevated by 7 days. PRL did not alter *Th* promoter activity in CAD cells, and STAT5B did not bind three putative Gamma Interferon Activation Sites (GAS) elements. We conclude that PRL initiates an integrated cascade of cellular mechanisms in TIDA neurons, including JAK2-STAT5B activation, TH Ser40 phosphorylation coupled to increased TH activity, followed by a delayed rise in *Th* gene expression. PRL-induced changes in *Th* gene expression are not the result of STAT5-mediated transactivation but likely result from enduring changes in TIDA neuronal activity.

## 1. Introduction

Dopamine is the major regulator of anterior pituitary prolactin (PRL) release [1,2,3,4,5]. Tuberoinfundibular dopaminergic (TIDA) neurons with cell bodies in the arcuate nucleus of the hypothalamus synthesize and secrete dopamine at the level of the nerve terminals in the median eminence (ME). Dopamine is then transported via the hypophysial portal blood to the anterior pituitary gland, where it binds dopamine D2 receptors on lactotrophs and inhibits PRL secretion. In addition to the well-established role of TIDA neurons in the suppression of PRL secretion, subpopulations of TIDA neurons may participate in other hypothalamic functions [6].

Tyrosine hydroxylase (TH) catalyzes the hydroxylation of L-tyrosine to 3,4-dihydroxyphenylalanine (DOPA) and is the rate-limiting step in dopamine biosynthesis. TH is regulated, in part, by both short-term phosphorylation state changes and long-term regulation of *Th* gene expression [7,8,9]. Most studies evaluate changes in *Th* mRNA levels, but some studies have identified transcriptional regulatory elements in the 5′ region of the *Th* gene [7]. TH is phosphorylated within an N-terminal regulatory domain on four conserved serine residues: Ser8, Ser19, Ser31 and Ser40. Phosphorylation of Ser40 and Ser31 increases TH activity, while phosphorylation of Ser19 increases Ser40 phosphorylation [7,8]. A number of protein kinases and phosphoprotein phosphatases are able to regulate the phosphorylation states of these serine residues, and some have been implicated in the regulation of TH activity in TIDA neurons [10,11,12,13,14].

PRL acts by short-loop negative feedback to regulate its secretion by triggering an increase in TIDA neuronal activity, but connections among cellular events and intracellular signaling pathways are incompletely understood [1,3,15,16,17]. Both the short and long forms of the PRL receptor (PRLR) are expressed in the arcuate nucleus, but the long form is predominant [18]. PRLR is co-localized with TH in hypothalamic dopaminergic neurons, although other non-dopaminergic hypothalamic cells also co-localize PRLR [13,19,20]. TIDA neurons involved in the PRL short loop feedback and the control of PRL secretion from the anterior pituitary are a subpopulation of TIDA neurons, which do not contain gamma-aminobutyric acid [21]. Demarest et al. [22] observed both a rapid ‘tonic’ and a delayed ‘induction’ component for PRL-induced activation of TIDA neurons, suggesting that multiple cellular mechanisms play a role. When basal circulating PRL levels are reduced by bromocriptine treatment, protein dephosphorylation is a mechanism for the acute 4 h suppression of TH activity, suggesting that low basal levels of PRL maintain TH in a phosphorylated state [11]. Moreover, PRL treatment increases both TH activity and TH phosphorylation at Ser19, Ser31, and Ser40 residues in embryonic hypothalamic cultures [13]. Inhibitors of signaling pathways that are implicated in the phosphorylation of these regulatory serines in TH reverse or prevent the PRL-induced increase in TH activity, suggesting a role of these membrane receptor-associated signaling pathways for the PRL response in TIDA neurons [12,13].

PRL binding to the long form of the PRLR activates the Janus Kinase 2 (JAK2)- Signal Transducer and Activator of Transcription 5 (STAT5) signaling pathway [23,24,25,26]. Nuclear STAT5B can bind to the palindromic STAT5-responsive gamma interferon activation site (GAS) element TTCNNNGAA and modulate transcription rates of specific target genes [24,25]. Under some circumstances, other intracellular signaling pathways may also be activated, most notably the mitogen-activated protein kinase (MAPK) pathway, Src kinases and phosphatidylinositol 3-kinase pathway [5,6,23,24]. Hyperprolactinemia increases, and bromocriptine-induced hypoprolactinemia decreases *Th* mRNA expression in the arcuate nucleus [16], indicating that PRL modulates *Th* gene expression. PRL increases nuclear translocation and phosphorylation of the STAT5B isoform in TIDA neurons [27,28,29], indicating the PRLR-JAK2-STAT5B pathway is activated in TIDA neurons. Mice deficient in STAT5B throughout development and lifespan show decreased levels of *Th* mRNA, and phosphorylated forms of STAT5 are not detected in the hypothalami of these mice, even though STAT5A is present [28,30].

The focus of this study was to evaluate time-dependent changes in JAK2-STAT5B signaling, TH activity, *Th* mRNA levels, and TH phosphorylation state in ovariectomized female rats treated with exogenous ovine PRL (oPRL). The direct influence of the PRLR-JAK2-STAT5B signaling pathway on *Th* promoter activity was also explored. The objectives of this study were to (1) determine time-dependent changes in JAK2-STAT5 phosphorylation state in the mediobasal hypothalamus (MBH) after injection of oPRL, (2) evaluate direct influences of PRLR-STAT5B signaling on *Th* promoter activity and binding of STAT5B to putative GAS elements in the 5′ regulatory region of the *Th* gene, (3) examine time-dependent changes in TH catalytic activity, TH phosphorylation state at Ser19, Ser31 and Ser40 and *Th* mRNA levels in TIDA neurons under conditions of hyperprolactinemia and (4) correlate TIDA neuronal activity changes with endogenous circulating PRL levels.

## 2. Materials and Methods

### 2.1. Animals and Hormone/Drug Treatments

**Figure 1:**
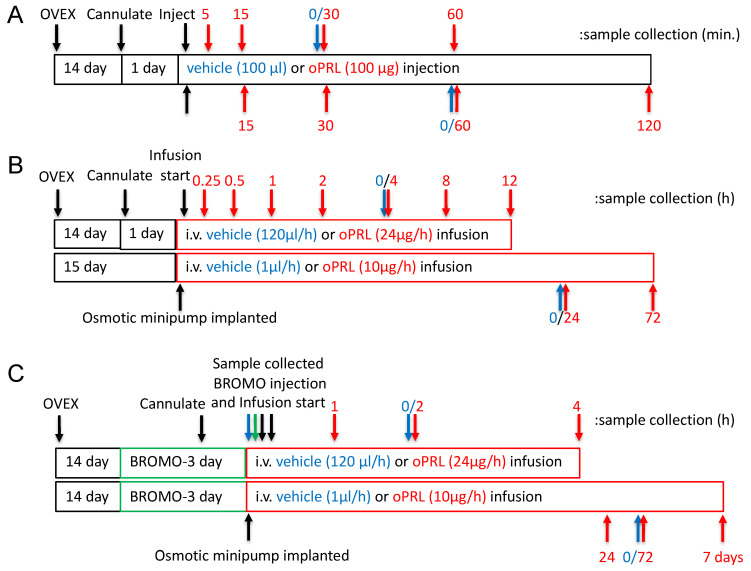
Experimental design timelines for procedures, hormone treatment and sample collection. (**A**) The timeline for experiments examining the JAK2 and STAT5 phosphorylation state in the MBH. The top arrows represent sample collection times for JAK2 phosphorylation studies, and the bottom arrows represent sample collection times for STAT5 phosphorylation. Black arrows indicate procedures, red arrows indicate sample collection for the oPRL-treated group, and blue arrows indicate sample collection for the vehicle-treated group (0 h with respect to oPRL treatment). (**B**) Experimental design timeline for circulating oPRL, circulating rPRL, TH activity in the ME, *Th* mRNA levels in the arcuate nucleus, and TH phosphorylation state in the ME after vehicle or oPRL treatment. The *top* bar and arrows represent treatment and sample collection times, respectively, for infusion studies of ≤12 h. The bottom bar and arrows represent treatment and sample collection times, respectively, for infusion studies of >12 h. Black arrows indicate procedures, red arrows indicate sample collection for the oPRL-treated group, and blue arrows indicate sample collection for the vehicle-treated group (0 h with respect to oPRL treatment). (**C**) The timeline for *Th* mRNA levels in the arcuate nucleus after oPRL treatment in vehicle or bromocriptine (BROMO) pre-treated rats. The top bar and arrows represent treatment and sample collection times, respectively, for infusion studies of ≤12 h. The bottom bar and arrows represent treatment and sample collection times, respectively, for infusion studies of >12 h. Green bars represent 3 days of treatment with bromocriptine (BROMO) or vehicle with injections at 12 h intervals. Black arrows indicate procedures, red arrows indicate sample collection for the oPRL-treated group, blue arrows indicate sample collection for the vehicle-treated group (0 h with respect to oPRL treatment), and green arrows indicate sample collection for vehicle and bromocriptine (BROMO) treated groups.

Adult female (200–250 g; 2–3 months of age) Sprague–Dawley rats (Charles River, Raleigh, NC, USA) were used. Rats were housed under controlled temperature and lighting (lights on from 0700 h to 2100 h) and supplied with food and water ad libitum. Rats were administered meloxicam orally (1 mg/kg, p.o.) 1 h before surgery and 1-day post-surgery, but were discontinued 24 h before the experiment. All surgeries were performed under isoflurane (2–5% mixed with oxygen) anesthesia. Rats were ovariectomized 14 days before the experiment and implanted with a jugular cannula one day before oPRL injection experiments and oPRL infusion experiments of ≤12 h (Figure 1A,B). The jugular cannula was exteriorized at the dorsal neck. For oPRL injection experiments, rats were injected intravenously (i.v.) with 100 µg oPRL or 100 µL vehicle (0.01 M sodium bicarbonate, pH 8.6, 0.15 M sodium chloride) and euthanized at specified times after the injection (Figure 1A). For ≤12 h oPRL infusion experiments, oPRL was initially solubilized to a concentration of 1 mg/mL in 0.01 M sodium bicarbonate, pH 8.6, 0.15 M sodium chloride, 50 U/mL heparin, and then diluted to 200 µg/mL with heparinized saline (0.15 M sodium chloride, 20 U/mL heparin). Rats were given an initial 50 µL bolus injection of 10 µg oPRL or vehicle and then were constantly infused with 200 µg/mL oPRL or vehicle at a rate of 120 µL/hour using an Ismatec peristaltic pump (Figure 1B,C). For ≥24 h of oPRL infusion, rats were implanted with a chronic jugular cannula on the day of Alzet osmotic minipump implantation (Figure 1B,C). The osmotic minipumps filled with oPRL (10 mg/mL) were connected to the jugular cannula and secured subcutaneously (s.c.) on the ventral chest. oPRL was delivered with a flow rate of 1 µL/h for up to 3 days (Model 1003D) or for 7 days (Model 2001). Where indicated, some rats were injected subcutaneously (s.c.) with bromocriptine mesylate (3 mg/kg; Tocris Biosciences, Minneapolis, MN USA) or 20% ethanol vehicle (0.5 mL/kg rat weight, s.c.) at 12 h intervals (Figure 1C). All animal experiments were conducted according to the National Institutes of Health Guide for the Care and Use of Laboratory Animals and approved by the Institutional Animal Care and Use Committee at Southern Illinois University at Carbondale.

### 2.2. Cell Lines

CAD cells (gift of Dr. James T. K. Wang Tufts University, Boston, MA, USA) were maintained in DMEM:F-12 (1:1) media supplemented with 8% fetal bovine serum (FBS) and penicillin/streptomycin and have been described previously [31]. PC-12 cells (ATCC, Manassas, VA, USA) were maintained in RPMI media supplemented with 10% horse serum, 5% FBS, and penicillin/streptomycin. N-39 cells (CELLutions Biosystems Inc., Burlington, NC, USA) were maintained in DMEM supplemented with 10% FBS and penicillin/streptomycin. All cells were maintained at 37 °C in 5% carbon dioxide.

### 2.3. Immunoprecipitation

For JAK2 and STAT5 phosphorylation analysis, the brain was rapidly removed after decapitation and frozen immediately in Histofreeze or Friendly Freeze (Fisher Scientific, St. Louis, MO, USA) at −80 °C. The MBH was excised from the frozen brain using a 2 mm diameter sample corer with a plunger (Fine Science Tools, Foster City, CA, USA), and a fragment 1.75 mm deep was used for analysis. The tissue fragment was homogenized in a homogenization/immunoprecipitation buffer containing protease and phosphatase inhibitors, as described previously [32,33]. Equivalent protein amounts of the MBH samples were precleared with protein G Plus/A agarose (Calbiochem, Burlington, MA, USA). The samples were incubated for 18 h at 4 °C with 2 µL/ sample anti-STAT5 (C-17, Santa Cruz Biotechnology, Dallas, TX, USA), anti-STAT5A (L-20, Santa Cruz Biotechnology), anti-STAT5B (G-2, Santa Cruz Biotechnology) or anti-JAK2 (Upstate Biotechnology, Lake Placid, NY, USA). The antibody-protein complex was precipitated with protein G Plus/A agarose (Calbiochem) or Dynabeads protein G (Invitrogen, Carlsbad, CA, USA), and the precipitate was washed four times. Protein samples were prepared in reducing Laemmli sample buffer and heated at 95 °C for 4 min. Samples were centrifuged at 10,000× *g* for 10 min, and supernatant was used for western blot analysis.

### 2.4. Western Blots

For time analysis of JAK2 and STAT5 phosphorylation state, immunoprecipitated MBH samples were separated by sodium dodecyl sulfate-polyacrylamide gel electrophoresis (SDS-PAGE), and proteins were transferred to Immobilon P polyvinylidene fluoride (PVDF) membrane and subjected to western blot analysis. Non-specific binding was blocked by incubation with 5% bovine serum albumin (BSA) at room temperature for 1 h. For JAK2 and STAT5 phosphorylation time course experiments, blots were incubated with rabbit anti-pSTAT5 Tyr694 (1:2000; Upstate Biotechnology) or rabbit anti-pJAK2 Tyr 1007 Tyr 1008 (1:3000; Upstate Biotechnology) for 18 h at 4 °C, followed by horseradish peroxidase (HRP)-conjugated anti-rabbit IgG (1:2000) for 1 h at room temperature. Blots were stripped and subsequently incubated with rabbit anti-STAT5 (C-17 1:2000; Santa Cruz Biotechnology) or rabbit anti-JAK2 (C-20 1:2500; Santa Cruz Biotechnology) followed by HRP-conjugated anti-rabbit IgG (1:2000). For analysis of total STAT5 and JAK2, 25 µg protein of input MBH from samples above was separated by SDS-PAGE and proteins transferred to a second set of Immobilon P membranes and subjected to western blot analysis. Blots were incubated with rabbit anti-STAT5 (C-17 1:2000) or rabbit anti-JAK2 (C-20 1:2500), followed by HRP-conjugated anti-rabbit IgG (1:2000). Blots were stripped and incubated with mouse anti-β-tubulin (1:4000, Upstate Biotechnology) followed by HRP-conjugated anti-mouse IgG (1:2000). Individual protein signals were visualized using ECL enhanced luminol reagent (Amersham Pharmacia, Piscataway, NJ, USA) and detected using film. The signal volumes for protein bands of interest were quantified using a Molecular Dynamics densitometer. The density signal of phospho-JAK2 (pJAK2) and phospho-STAT5 (pSTAT5) was normalized to total JAK2 and STAT5, respectively, for each sample, and then experimental groups were calculated as percent control signal on the same blot. The density signal of JAK2 and STAT5 was normalized to β-tubulin, and then experimental groups were calculated as a percentage of the control signal on the same blot.

For analysis of the STAT5 isoform phosphorylation state, immunoprecipitated MBH samples were separated by SDS-PAGE, and proteins were transferred to Immobilon P PVDF membrane and subjected to western blot analysis. Non-specific binding was blocked with 0.2% I-Block (LI-COR, Lincoln, NE, USA) and then blots were incubated with rabbit anti-pSTAT5 Tyr694 (1:2000; Upstate Biotechnology), rabbit anti-STAT5 (C-17 1:2000; Santa Cruz Biotechnology), rabbit anti-STAT5A (L-20, Santa Cruz Biotechnology) and/or mouse anti-STAT5B (G-2 1:2000; Santa Cruz Biotechnology), followed by Alexa Flour 680 goat anti-mouse IgG (1:20,000; Fisher Scientific) and/or IR dye 800 goat anti-rabbit IgG (1:20,000; LI-COR). Individual protein signals were visualized using LI-COR Odyssey Imager (LI-COR, Lincoln, NE, USA).

Transfected cells were collected in a lysis buffer [34]. Fifteen µg of protein lysate were separated on SDS-PAGE gels and subjected to western blot analysis. Blots were incubated with rabbit anti-pSTAT5 Tyr694 (1:2000; Upstate Biotechnology) and mouse anti-STAT5B (G-2, 1:2000; Santa Cruz Biotechnology), followed by Alexa Flour 680 goat anti-mouse IgG (1:20,000; Fisher Scientific) and/or IR dye 800 goat anti-rabbit IgG (1:20,000; LI-COR). Individual protein signals were visualized using LI-COR Odyssey Imager.

For TH phosphorylation analysis, the brain was rapidly removed after decapitation, and the ME was dissected with fine scissors from the ventral surface of the brain under a dissecting microscope. The ME was homogenized in 35 µL homogenization buffer described previously [32], and proteins were separated by SDS-PAGE and subjected to western blot analysis. Fifteen micrograms of protein were loaded per well for TH protein, phospho-TH (pTH)Ser40 and pTHSer19 analysis, whereas 30 µg protein was loaded for pTHSer31 analysis. For blots with 0.25, 0.5, 1, 2, 4, 24, and 72 h time points, non-specific binding was blocked with 5% BSA. Blots for pTH analysis were incubated with rabbit anti-pTHSer40 (1:2500; Zymed, San Francisco, CA, USA), rabbit anti-pTHSer31 (1:2500; Calbiochem), or rabbit anti-pTHSer19 (1:2500; Oncogene, Cambridge, MA, USA), followed by HRP-conjugated anti-rabbit IgG. Blots were stripped and then incubated with mouse anti-TH (1:3000; Chemicon, Temecula, CA, USA), followed by HRP-conjugated anti-mouse IgG. Individual protein signals were visualized using ECL enhanced luminol reagent (Amersham Pharmacia) and detected using film. The signal volumes for protein bands of interest were quantified using a Molecular Dynamics densitometer. For blots with 4 h, 8 h, 12 h, 24 h, and 72 h time points, non-specific binding was blocked with 0.2% I-Block. For pTH analysis, membranes were incubated with rabbit anti-pTHSer40 (1:2500; Calbiochem), rabbit anti-pTHSer31 (1:2500, Calbiochem), or rabbit anti-pTHSer19 (1:2500; Calbiochem) together with mouse anti-TH (1:3000; Chemicon). Subsequently, blots were incubated with AlexaFlour 680 goat anti-mouse IgG (1:20,000; Fisher Scientific) and/or IR dye 800 goat anti-rabbit IgG (1:20,000; LI-COR). Individual protein signals were visualized using LI-COR Odyssey Imager. Individual protein signals were visualized using LI-COR Odyssey Imager, and the fluorescence signals for protein bands of interest were quantified separately in the 680 nm and 800 nm channels using the associated Image Studio V2.1.10 analysis software. The density signal of pTHSer40, pTHSer31 and pTHSer19 was normalized to total TH for each sample, and then experimental groups were calculated as a percent of the control signal on the same blot. The density signal of TH was normalized to β-tubulin, and then experimental groups were calculated as a percentage of the control signal on the same blot.

### 2.5. Plasmids

The open reading frame of rat PRLR was amplified by polymerase chain reaction (PCR) and inserted into pcDNA3 [34]. FLAG-STAT5B was generated by PCR amplification of the STAT5B open reading frame using primers that contained restriction sites that would allow for in-frame ligation into an N-terminal FLAG-tagged plasmid [34]. The FLAG-STAT5B-N642H mutant was generated by PCR. *Th* promoter-luciferase constructs have been previously described [33]. The rat casein promoter (−344 bp to −1 bp) was cloned from rat genomic deoxyribonucleic acid (DNA) by PCR and ligated into a pGL3 basic vector (Promega, Madison, WI, USA) luciferase construct. The HA-nuclear receptor-related 1 (NURR1) construct was isolated by PCR amplification of the open reading frame from reverse-transcribed PC-12 cell RNA. Primers contained restriction enzyme sites to allow in-frame ligation into an N-terminal tagged vector. All constructs were verified by DNA sequencing on a Beckman Coulter CEQ 8000.

### 2.6. Cell Transfection and Luciferase Assay

Cells (75,000 cells/ well) on 24 well plates were transfected with 500 ng PRLR, 500 ng STAT5B, 500 ng *Th* promoter and 25 ng cytomegalovirus (CMV)-Renilla luciferase expression constructs using the calcium phosphate technique for CAD and N-39 cells and by Fugene 6 for PC-12 cells following manufacturer’s protocols. Eighteen hours later, the media was replaced with serum-free media containing 1% Insulin-Transferrin-Selenium (ITS) premix and pen/strep or fresh media. Four hours later, the cells were treated with 1000 ng/mL oPRL for 24 h. Luciferase assays were performed with the Promega Dual-Luciferase Reporter Assay System following the manufacturer’s protocol using a Bio-Tek Clarity dual injector luminometer.

### 2.7. Immunofluorescence

CAD cells (400,000 cells/35 mm dish) were transfected with 2.5 µg of PRLR and 2.5 µg of STAT5B expression plasmids. Cells were fixed in 4% paraformaldehyde and incubated with rabbit anti-pSTAT5 (1:2000; Upstate Biotechnology) and mouse anti-STAT5B (G-2, 1:2000; Santa Cruz Biotechnology), followed by AlexaFluor596 anti-rabbit IgG and AlexaFluor488 anti-mouse IgG. DNA was stained with Hoescht dye 33,258 (1 µg/mL). Cells were visualized with an Olympus BW50 fluorescence microscope (Olympus, Tokyo, Japan) using a ×60 water objective. 

### 2.8. Recombinant Protein Isolation and Electrophoretic Mobility Shift Assay (EMSA)

CAD cells on 100 mM plates were transfected with 10 µg of FLAG-STAT5B or FLAG-STAT5B-N642H plasmid DNA for 18 h. Twenty-four hours after transfection, cells were lysed in a buffer containing 150 mM sodium chloride, 50 mM Tris (pH = 7.5), 1.0% Triton X-100, 1.0 mM ethylenediaminetetraacetic acid (EDTA), 1 mM sodium fluoride, 0.2 mM sodium orthovanadate, aprotinin (10 µg/mL), leupeptin (10 µg/mL) and pepstatin A (10 µg/mL). Lysates were applied to M2 FLAG agarose beads (Sigma Aldrich, St. Louis, MO, USA) and incubated overnight at 4 °C. Beads were then washed 5 times with lysis buffer and once with Tris-buffered saline (TBS, pH = 7.5). Proteins were eluted in TBS with 200 ng/mL FLAG peptide. Some of the purified proteins were then separated on SDS-PAGE and either stained with Coomassie Blue or subjected to western blot analysis using anti-STAT5 and anti-pSTAT5 antibodies. Radiolabeled double-stranded DNA (dsDNA) probes were generated by PCR using ^32^P adenosine triphosphate (ATP). Primer sequences used to generate the probes are shown below. Purified proteins were incubated in EMSA buffer (50 mM potassium chloride, 10 mM Tris pH = 7.5, 1 mM EDTA, 5.0% glycerol, 0.05% IGEPAL 630, and 1 mM dithiothreitol (DTT) with radiolabeled dsDNA probes for 20 min at room temperature, and reactions were then electrophoresed on 5% nondenaturing acrylamide gels and visualized on a Storm Phosphoimager.

Control for 5′-TGGACTTCTTGGAATTAAGGG-3′

Control rev 5′-TCCCTTAATTCCAAGAAGTCC-3′

*Cish* for 5′-TGCGGCTTCCGGGAAGGGCT-3′

*Cish* rev 5′-TAGCCCTTCCCGGAAGCCGC-3′

*Th* GAS 3 for 5′-TAGCGCTTCAGAGAAGCCTG-3′

*Th* GAS 3 rev 5′-TCAGGCTTCTCTGAAGCGCT-3′

*Th* GAS 2 for 5′-TCTGTCTTCCTTGAAGACAG-3′

*Th* GAS 2 rev 5′-TCTGTCTTCAAGGAAGACAG-3′

*Th* GAS 1 for 5′-TCACTTTTCTCTGAAGGGCT-3′

*Th* GAS 1 rev 5′-TAGCCCTTCAGAGAAAAGTG-3′

*Th* GAS 1intmut for 5′-TCACTTTTCT**TG**GAAGGGCT-3′

*Th* GAS 1intmut rev 5′-TAGCCCTTCCAAGAAAAGTG-3′

### 2.9. In Situ Hybridization

The method for in situ hybridization has been described previously [16,35]. Brains were quickly removed after decapitation, frozen in Friendly Freeze (Fisher Scientific), and stored at −80 °C. Coronal brain sections (20 µm) were cut through the arcuate nucleus. Alternate sections were used for the in situ hybridization procedure. After fixation in 4% paraformaldehyde and prehybridization steps described previously [16], the sections were hybridized for 4 h at 45 °C with a 50 ng/mL ^35^S-labeled complementary RNA (cRNA) probe. The antisense cRNA probe was transcribed from 1.1 kb BamHI/EcoRI TH complementary DNA (cDNA), inserted, and subcloned into a pSP65 vector, which was linearized with BamHI. The ^35^S-labeled cRNA was synthesized using SP6 RNA polymerase and [α-^35^S] guanosine triphosphate (GTP). The labeled probe had a specific activity of 5 × 10^8^ disintegrations per minute (dpm)/µg. After ribonuclease treatment and a series of post-hybridization washes that increased in stringency, the slides were dipped in Ilford K-5 Emulsion diluted 0.25 g/mL water. The autoradiograms were exposed for 3 weeks and developed by standard photographic methods. Brain sections were post-stained lightly with hematoxylin to visualize the cell nucleus.

Twenty alternate sections per animal were used for quantification of *Th* mRNA signal levels throughout the arcuate nucleus from −2.5 to −3.1 mm relative to bregma, identified using a rat brain atlas [36]. Analysis was performed by a person blinded to any expected outcomes for the experiment. *Th* mRNA-containing cells were identified under darkfield optics as a cluster of reduced silver grains with an identifiable cell nucleus. A threshold was set for the entire experiment, and the thresholded gray area for each cell was measured under ×400 darkfield illumination using NIH Image J v1.61. The number of silver grains in individual mRNA-containing cells was calculated. The mean grain area per cell was first calculated for individual animals. The individual animal means were used to calculate the mean ± SE of each experimental group and for statistical analysis.

### 2.10. Quantitative Reverse Transcriptase-Polymerase Chain Reaction (qRT-PCR)

Brains were quickly removed after decapitation, frozen in Friendly Freeze (Fisher Scientific), and stored at −80 °C. Four 300 µm brain sections containing the arcuate nucleus (−2.1 to −3.3 mm relative to bregma) were sliced in a cryostat at −9 °C. Sections were cooled to −20 °C on slides, and a region of the hypothalamus containing the arcuate nucleus was micro-dissected using a 1.5 mm biopsy punch (Miltex). RNA was isolated from micro-dissected tissue using Trizol reagent. Five hundred ng of RNA was reverse transcribed, and cDNA was analyzed by quantitative PCR using the following primer sets:

*Th* forward 5′-ACCGCACATTTGCCCAGTTC-3′

*Th* reverse 5′-GCTCCCCATTCTGTTTACATAGCC-3′

*Gapdh* forward 5′AACGACCCCTTCATTGACC-3′

*Gapdh* reverse 3′-TCCACGACATACTCAGCAC-3′

Fold expression was quantified by the 2^−ΔΔCT^ method.

### 2.11. DOPA Accumulation

Rats were implanted with a chronic jugular cannula on the day before the experiment and injected with m-hydroxybenzylhydrazine dihydrochloride (NSD1015; 25 mg/kg i.v.) after completion of vehicle or oPRL injection/infusion on the day of the experiment. Fifteen minutes thereafter, brains were rapidly removed after decapitation, and the ME was dissected with fine scissors under a dissection microscope. The ME was sonicated in 120 µL 0.1 N perchloric acid and centrifuged at 10,000× *g* for 2 min DOPA content in the ME was determined by high-performance liquid chromatography (HPLC) with electrochemical detection, as described previously [32]. The pellet was solubilized in 0.5 N sodium hydroxide and analyzed for protein content using the Bio-Rad Protein Assay.

### 2.12. PRL Assays

Trunk blood was collected at the end of the experiment. Blood was centrifuged at 10,000× *g* for 5 min, and serum oPRL and rat PRL (rPRL) were assessed using RIA kits provided by Dr. Albert Parlow and the National Hormone and Pituitary Program (Harbor-UCLA Medical Center, Los Angeles, CA, USA). The reference preparation for rPRL was RP-3, and the sensitivity limit for the assay was 0.25 ng/mL. The intra- and inter-assay coefficients of variation were 7.6% and 9.3%, respectively. The reference preparation for oPRL was I-3, and the sensitivity limit for the assay was 1.25 ng/mL. The intra- and inter-assay coefficients of variation were 4.1% and 1.2%, respectively.

### 2.13. Statistics

Results are expressed as mean ± SE. The n for all experiments refers to the number of experimental animals. Data were evaluated by ANOVA followed by Fisher’s least significant post-hoc test. When only 2 groups were compared, means were compared using Student’s *t*-test. *p* < 0.05 was considered statistically significant.

## 3. Results

### 3.1. Effect of oPRL on STAT5 and JAK2 Phosphorylation

**Figure 2:**
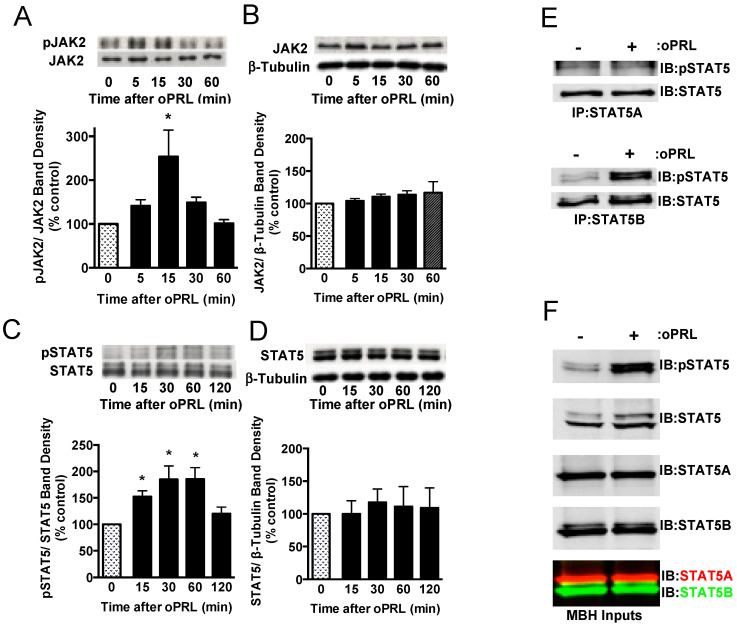
Time course for PRLR activation of JAK2/STAT5 pathway in the mediobasal hypothalamus (MBH) of ovariectomized female rats injected with oPRL (100 µg/rat, i.v.). MBH lysates were used for western blot analysis. (**A**) Top panel: Representative western blot of pJAK2 and JAK2 protein signals. Bottom panel: pJAK2 band density signal normalized to JAK2 band density signal and expressed as percent control. Each value is a mean ± SE of determinations from 6 rats. (**B**) Top panel: Representative western blot of JAK2 and β-tubulin protein signals. Bottom panel: Total JAK2 protein band density signal normalized to β-tubulin band density signal and expressed as percent control. Each value is a mean ± SE of determinations from 6 rats. (**C**) Top panel: Representative western blot of pSTAT5 and STAT5 protein signals. Bottom panel: pSTAT5 band density signal normalized to STAT5 band density signal and expressed as percent control. Each value is a mean ± SE of determinations from 8 rats. (**D**) Top panel: Representative western blot of STAT5 and β-tubulin. Bottom panel: Total STAT5 protein band density signal normalized to β-tubulin band density signal and expressed as percent control. Each value is a mean ± SE of determinations from 8 rats. (**A**–**D**) Stippled bars are samples from rats infused with vehicle and black bars are samples from oPRL-treated rats, as indicated in Figure 1A. (**E**) A representative western blot of MBH lysates was first immunoprecipitated with selective STAT5A (Top panel) or STAT5B (Bottom panel) antibodies and then blotted with pSTAT5 and pan-STAT5 antibodies. MBH tissues were collected at 45 min after oPRL or vehicle injection. (**F**) Representative western blots of MBH input lysates from above are blotted with the indicated antibodies. * Significantly different (*p* < 0.05) from the control group.

PRL is able to activate the PRLR-JAK2-STAT5 signaling pathway in hypothalamic dopaminergic neurons [27,28,29]. Experiments were carried out in ovariectomized rats given a single bolus injection of 100 µg oPRL to confirm the activation of the JAK2-STAT5 pathway in the MBH and analyze the changes in the phosphorylation state of immunoprecipitated STAT5 and JAK2 at various time points after oPRL administration (Figure 1A). Circulating oPRL levels were assessed from trunk blood in a separate group of rats (n = 4). oPRL was markedly increased to 3576 ± 176 ng/mL within 5 min after the i.v. injection and subsequently declined to 725 ± 157, 239 ± 31, 75 ± 20, and 22 ± 5 ng/mL at 15, 30, 60, and 120 min, respectively. Phosphorylated JAK2 levels were significantly increased at 15 min and then returned to basal levels at 30 min (Figure 2A). No changes in total JAK2 protein (normalized to β-tubulin protein levels) were detected (Figure 2B). Phosphorylated STAT5 protein levels increased at 15 min and peaked at 30–60 min after oPRL injection. STAT5 phosphorylation levels returned to basal levels by 2 h (Figure 2C). No changes in total STAT5 protein levels (normalized to β-tubulin protein levels) were detected (Figure 2D).

At 45 min after a single injection of vehicle or oPRL, immunoprecipitations were performed on MBH lysates using antibodies specific to either STAT5A or STAT5B to determine which STAT5 isoform was phosphorylated with oPRL treatment. No changes in phosphorylated STAT5A were observed with oPRL treatment; however, phosphorylated STAT5B levels increased with oPRL treatment (Figure 2E). Western blots were performed on the MBH inputs used for immunoprecipitations to confirm the increase in phosphorylated STAT5B with oPRL treatment and also the expression of STAT5A and STAT5B in the MBH (Figure 2F). These blots also showed the specificities of the STAT5A and STAT5B antibodies (bottom panel, Figure 2F).

### 3.2. PRLR-STAT5B Signaling to the TH Promoter

**Figure 3:**
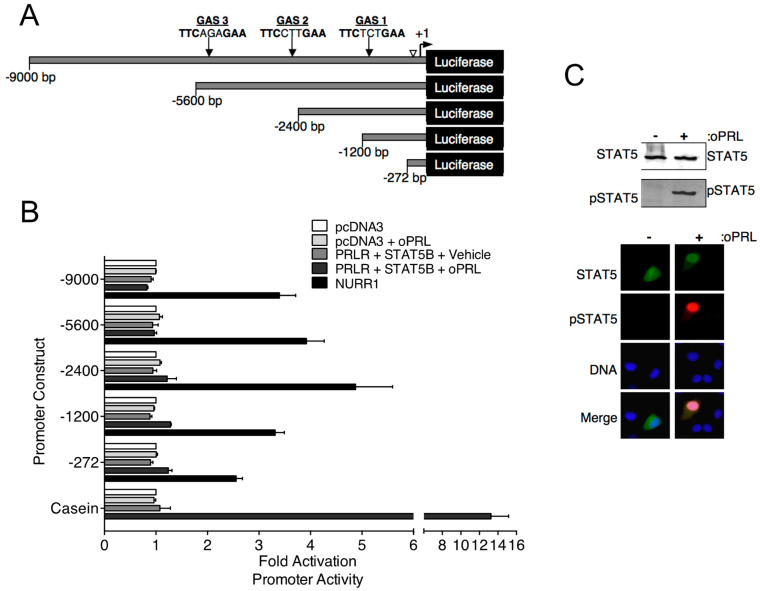
Effects of PRL-STAT5B signaling on rat *Th* promoter constructs. (**A**) Schematic of rat *Th* promoter used in luciferase assays. Numbers indicate the size of constructs, and arrows indicate the location of potential STAT5 binding sequences or gamma interferon-activated sites (GAS). An open arrowhead indicates a NRBE binding site for nuclear receptor-related 1 (NURR1) protein, which was used as a positive control for *Th* promoter activity. (**B**) CAD cells were co-transfected with the indicated *Th* promoter-luciferase or casein promoter-luciferase constructs as indicated and with pDNA3, CMV-rPRLR, CMV-rSTAT5B, or rNURR1 constructs as indicated. Cells were also co-transfected with CMV-renilla luciferase to determine transfection efficiency. Cells were treated with vehicle or oPRL (1000 ng/mL) for 24 h, followed by dual luciferase assays. Luciferase values were normalized to the pcDNA3+ vehicle values to determine fold activation. A −0.5 kb casein promoter fragment containing a GAS element was used as a control for PRL-driven promoter activation. Each value is a mean ± SE of 3 independent luciferase assays. (**C**) PRL-mediated STAT5B signaling in CAD cells. CAD cells were transfected with rPRLR and rSTAT5B and then treated with vehicle or oPRL (1000 ng/mL) for 1 h. Top panel: Cell lysates of the vehicle and oPRL-treated cells were used in western blot analysis with the indicated antibodies. Bottom panel: Immunofluorescence was used to detect the cellular localization of pSTAT5 (green) and pSTAT5 (red) in vehicle and oPRL-treated cells. DNA stain (blue) indicates cell nucleus.

Previous in vivo studies indicate that PRL treatment increases *Th* mRNA expression in the rat arcuate nucleus after 3 days of oPRL administration [16]. Experiments were designed to investigate the direct influence of PRLR-STAT5B signaling on *Th* promoter activity using the mouse neuronal *Th*-expressing CAD cell line. CAD cells were transfected with PRLR and STAT5B expression constructs. Cells were also transfected with the indicated *Th* promoter deletion constructs fused to the luciferase reporter gene (Figure 3A). Putative GAS sites in the *Th* promoter constructs were identified by sequence analysis (Figure 3A). Cells were treated with vehicle or 1000 ng/mL oPRL, followed by luciferase assays. Compared to vehicle-treated cells, PRL treatment had no influence on promoter activity for any of the *Th* promoter deletion constructs (Figure 3B). Positive control experiments were also performed to confirm that both PRLR-STAT5B signaling was activated with PRL treatment, and *Th* promoter activity could increase in CAD cells. The transcription factor NURR1 has been reported to increase *Th* promoter activity and was transfected instead of PRLR and STAT5B in a separate group of cells as a positive control. Cells transfected with NURR1 showed a 2.5- to 5.0-fold increase in *Th* promoter activity for all of the *Th* promoter deletion constructs (Figure 3B). The PRLR-STAT5B signaling pathway activates casein promoter activity. Therefore, a casein promoter construct was transfected instead of the *Th* promoter construct in a separate group of cells to show that PRLR-STAT5B signaling was activated in these cells. Compared to vehicle-treated cells, oPRL treatment increased casein promoter activity 13-fold (Figure 3B). Immunofluorescence and western blot experiments were performed on CAD cells transfected with PRLR and STAT5B and treated with vehicle or PRL for 1h to further confirm PRLR-STAT5B signaling activation. PRL treatment caused both an increase in STAT5B nuclear localization and tyrosine phosphorylation when compared to vehicle-treated cells (Figure 3C). Similar transcription assays were also performed in *Th* -positive rat pheochromocytoma PC-12 and hypothalamic mouse N-39 cell lines. PRL treatment of transfected PC-12 and N-39 cell lines did not increase the promoter activity of either the −2400 bp or the −272 bp *Th* promoter constructs (Figure S1). NURR1 increased *Th* promoter activity, and PRL treatment increased casein promoter activity in transfected PC-12 and N-39 cell lines.

**Figure 4:**
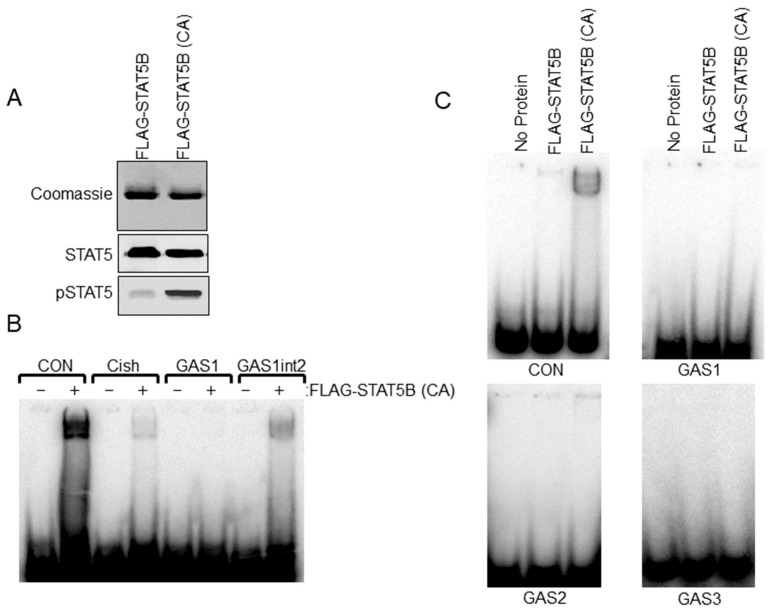
Electrophoretic Mobility Shift Assay (EMSA) analysis of STAT5B binding to putative gamma interferon activation site (GAS) elements in the *Th* promoter. (**A**) FLAG-STAT5B and constitutively active FLAG-STAT5B (CA) were purified from transfected HEK 293t cells. Purified proteins were separated on SDS-PAGE gels and stained with Coomassie Blue to detect proteins (top panel) or used in western blot analysis for Stat5 and pSTAT5 proteins (bottom panels) to confirm the purity of isolated protein and increased phosphorylation of FLAG-STAT5B (CA) compared to wildtype unphosphorylated FLAG-STAT5B. (**B**) EMSA analysis using FLAG-STAT5B (CA) protein and the indicated dsDNA probes for GAS sites for the control casein (CON) promoter (TGGACTTCTTGGAATTAAGGGT), *Cish* promoter, GAS1 site of the rat *Th* promoter at −1061 bp and the *Th* GAS1 site when the internal sites of the GAS1 site were mutated to those of the casein GAS site (GAS1int2). Note that the band indicates binding to CON, *Cish*, and GAS1int2 sequences but not the *Th* GAS1 site at −1061 bp. Sequences for the dsDNA probes are listed in the bottom panel. (**C**) EMSA analysis using wildtype unphosphorylated FLAG-STAT5B or FLAG-STAT5B (CA) and dsDNA probes for the −1061 bp (top right panel), −2751 bp (bottom left panel), and the −5121 bp (bottom right panel) putative GAS elements of the *Th* promoter. A control casein (CON) promoter sequence was used on the top left panel.

Sequence analysis of the rat −9000 bp *Th* promoter indicated three putative STAT5 response sites or GAS elements (5′-TTCNNNGAA-3′). Relative locations (−1061 bp, −2757 bp and −5121 bp) and sequences of these elements are shown in Figure 3A. We sought to further address the ability of STAT5B to interact with putative GAS elements within the *Th* promoter. Radiolabeled dsDNA probes were generated for each of the three GAS elements. Positive control GAS element sequences from the casein and *Cish* promoters were also generated. STAT5B and STAT5B-N642H proteins were purified from transfected cells and used for EMSA experiments. STAT5B-N642H protein is phosphorylated without cytokine pathway activation and shows increased levels of tyrosine 699 phosphorylation compared to wild-type STAT5 protein (Figure 4A). The dsDNA probes were incubated with no protein, STAT5B (not phosphorylated-negative control), or STAT5B-N642H (constitutively active) and analyzed by EMSA (Figure 4C). STAT5B (not active) did not bind any of the probes. STAT5B-N642H bound the casein control GAS element but did not bind to any of the putative *Th* GAS elements. STAT5B-N642H was also able to bind a GAS element from the *Cish* promoter (Figure 4B). Interestingly, STAT5B-N642H was able to bind to the first putative *Th* GAS element when two of the internal bases were mutated to those of the casein GAS element sequence (Figure 4B). These data indicate that specific internal nucleotides within the TTCNNNGAA sequence also influence STAT5-DNA interactions.

### 3.3. TH Activity in the ME of oPRL-Treated Rats

**Figure 5:**
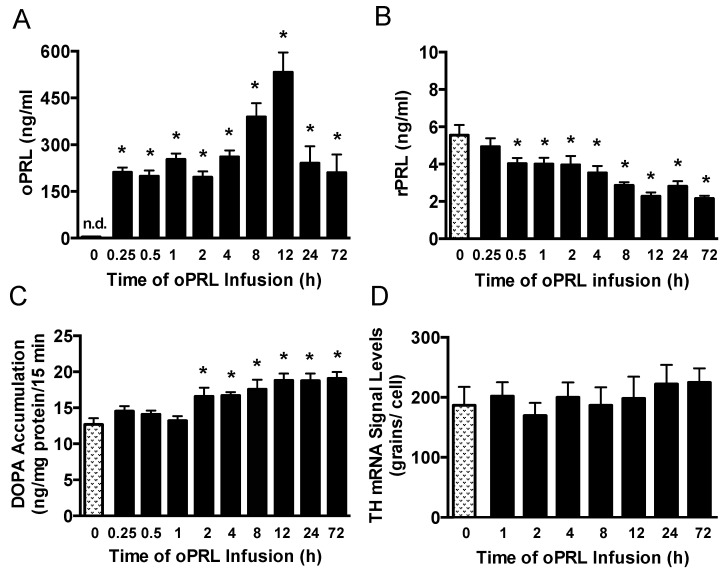
Time course of circulating endogenous rPRL levels, TH catalytic activity in the ME, and *Th* gene expression in the arcuate nucleus after the initiation of oPRL infusion. Serum oPRL levels (**A**) and endogenous serum rPRL levels (**B**) at indicated times after initiating oPRL infusion. oPRL (200 µg/mL) was infused at a rate of 120 µL/h for up to 12 h, and oPRL (10 mg/mL) was infused at a rate of 1 µL/h using an Alzet osmotic minipump for the 24 h and 72 h groups. Each value is a mean ± SE of determinations from 14 to 16 rats for the 0.25 h to 72 h group and 37 rats for the control (0 h) group. Note that PRL levels were determined from rats used for TH phosphorylation, and two control samples were included on each blot (Figure 6). (**C**) TH catalytic activity as assessed by dihydroxyphenylalanine (DOPA) accumulation in the ME at 15 min after injection of m-hydroxybenzylhydrazine dihydrochloride (NSD1015; 25 mg/kg i.v.). Each value is a mean ± SE of determinations from 9 to 10 rats. (**D**) *Th* gene expression as assessed by *Th* mRNA signal levels in the arcuate nucleus using the in situ hybridization technique. Each value is a mean ± SE of determinations from 5 to 7 rats. Stippled bars are samples from rats infused with vehicle and black bars are samples from oPRL-treated rats, as indicated in Figure 1B. * Significantly different (*p* < 0.05) from the control (0 h) group.

Experiments were performed to correlate changes in serum PRL levels with TH activity in the ME of ovariectomized rats constantly infused with oPRL at various times (Figure 1B). Serum oPRL and rPRL levels were assayed after oPRL infusion. Ovine PRL levels were elevated between 200 and 300 ng/mL at 0.25 h to 4 h after initiation of the infusion, but increased to 562 ng/mL at 12 h (Figure 5A). In rats infused using the osmotic minipumps for 24 h to 72 h, oPRL levels were 240 ng/mL. Infusion of oPRL significantly decreased rPRL serum levels by 0.5 h, and these levels remained attenuated between 1 h and 72 h (Figure 5B). It is notable that the decrease in rPRL levels showed a more profound decrease between 8 h and 72 h. TH activity was evaluated by DOPA accumulation in the ME of these rats. TH activity in the ME was unchanged at 0.25 h to 1 h after initiating oPRL infusion. A significant increase in DOPA accumulation was observed at 2 h, and DOPA accumulation remained elevated at 4 h, 8 h, 12 h, 24 h and 72 h (Figure 5C).

### 3.4. Effect of oPRL on TH mRNA Levels in the Arcuate Nucleus

We next evaluated PRL-mediated regulation of *Th* gene expression in the arcuate nucleus over the short time course when TH activity was elevated (Figure 1B). Ovariectomized rats were constantly infused with oPRL, and *Th* mRNA signal levels in the arcuate nucleus were evaluated at various times using in situ hybridization. Compared to 0 h control rats, oPRL infusion did not change *Th* mRNA signal levels in the arcuate nucleus between 1 h and 72 h (Figure 5D).

**Figure 6:**
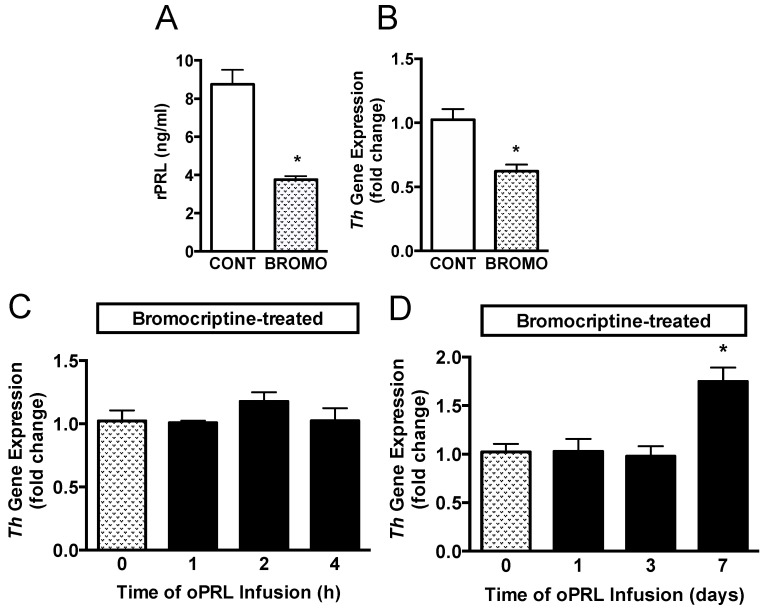
Time course for *Th* gene expression in the MBH in bromocriptine (BROMO)-treated rats with or without oPRL infusion. Endogenous serum rPRL levels (**A**) and *Th* gene expression as determined by qRT-PCR (**B**) in ovariectomized female rats after 3 days of bromocriptine (3 mg/kg, s.c.) or 20% ethanol vehicle control (CONT; 0.5 mL/kg rat weight, s.c.) treatment at 12 h intervals. Each value is a mean ± SE of determinations from 8 rats. White bars are samples from vehicle-treated (CONT) rats and stippled bars are samples from bromocriptine-treated (BROMO) rats. * Significantly different (*p* < 0.05) from the vehicle-treated control (CONT) group. (**C**,**D**) TH gene expression, as determined by qRT-PCR, in bromocriptine-treated ovariectomized female rats infused with oPRL (200 µg/mL) at a rate of 120 µL/h for up to 4 h (**C**) or infused with oPRL (10 mg/mL) at a rate of 1 µL/h using an Alzet osmotic minipump for 1 to 7 days (**D**). Each value is a mean ± SE of determinations from 8 rats. Stippled bars are samples from bromocriptine and vehicle-treated rats and black bars are samples from bromocriptine and oPRL-treated rats as indicated in Figure 1C. * Significantly different (*p* < 0.05) from the control (0 h) group.

Another group of ovariectomized rats was injected with vehicle or bromocriptine for 3 days to reduce basal rPRL levels to determine if endogenous rPRL has a role in sustaining *Th* mRNA levels (Figure 1C). Rat PRL (rPRL) was reduced to 3.5 ng/mL after 3 days of bromocriptine treatment (Figure 6A). *Th* mRNA expression in micro-dissected hypothalamic punches containing the arcuate nucleus was analyzed by qRT-PCR. *Th* mRNA expression was reduced by 40% after bromocriptine treatment (Figure 6B). A subsequent experiment evaluated whether short-term oPRL infusion could reverse the inhibitory effects of bromocriptine on *Th* mRNA expression. *Th* mRNA expression was unchanged with 1 h, 2 h, and 4 h of oPRL infusion (Figure 6C). Using osmotic minipumps, oPRL infusion was extended to 1, 3, and 7 days. Infusion of exogenous oPRL in bromocriptine-treated animals did not restore *Th* mRNA levels at 1 and 3 days (Figure 6D). However, a significant increase in *Th* mRNA was apparent after 7 days of oPRL infusion (Figure 6D).

### 3.5. Effect of oPRL on TH Phosphorylation State in the ME

**Figure 7:**
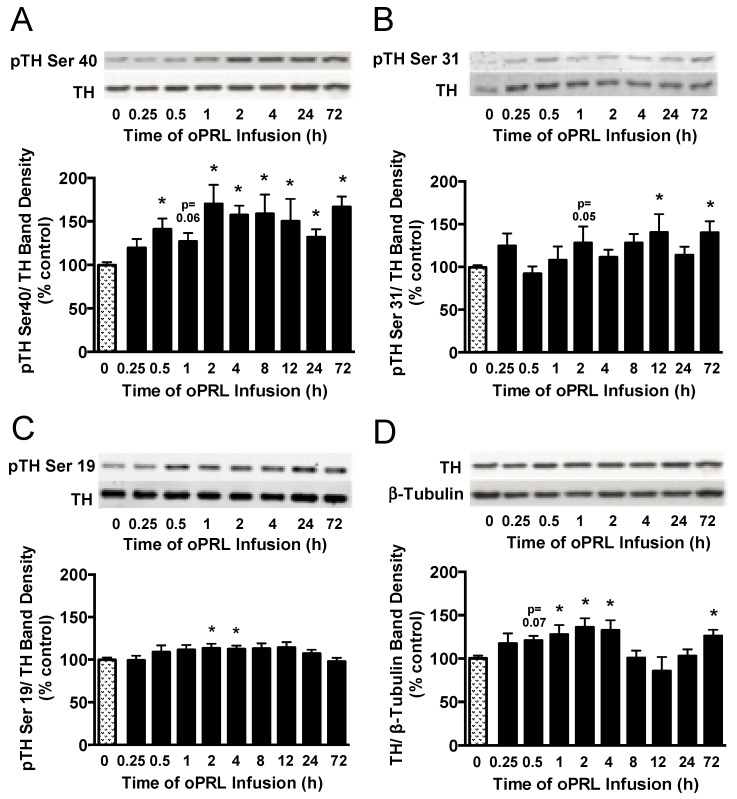
Time course for phosphorylation state of TH protein in the ME of ovariectomized female rats infused with oPRL (200 µg/h, i.v.) for the indicated time. ME lysates were used for western blot analysis of pSer40 (**A**), pSer31 (**B**), and pSer19 (**C**) in the regulatory domain of TH and total TH protein (**D**). (**A**–**C**) Top panels: Representative western blots of pTH at indicated serine residues and TH protein signals. Bottom panels: pTH band density signals at the indicated serine residue normalized to the respective TH band density signal on the same blot and expressed as percent control. (**D**) Top panel: Representative western blot of TH and β-tubulin protein signals. Bottom panel: Total TH protein band density signal normalized to β-tubulin band density signal and expressed as percent control. Each value is a mean ± SE of determinations from 7 to 15 rats for 0.25 h to 72 h and 37 to 39 rats for 0 h control samples. Note: two control samples were included on each blot and averaged before normalizing pTH values to the average 0 h control value for each blot. Given the limited number of lanes, not all time groups were included on the same blots, but all pTH or TH values were normalized to the average of two 0 h control values on the same blot. Stippled bars are samples from rats infused with vehicle and black bars are samples from oPRL-treated rats, as indicated in Figure 1B. * Significantly different (*p* < 0.05) from the control (0 h) group.

Next, we evaluated the phosphorylation state of the three N-terminal regulatory Ser sites in the TH protein that are associated with intracellular signaling pathways. TH phosphorylation states at Ser40, Ser31, and Ser19 were evaluated in the ME of ovariectomized rats constantly infused with oPRL (Figure 1B). TH Ser40 phosphorylation in the ME was significantly increased by 30–50% at 0.5 h, 2 h, 4 h, 8 h, 12 h, 24 h, and 72 h (Figure 7A), correlating with the increase in TH activity in the ME (Figure 5C). TH Ser31 phosphorylation levels showed significant increases at 12 h and 72 h (Figure 7B). Ser19 phosphorylation levels significantly increased at 2 h and 4 h (Figure 7C). Compared to 0 h control rats, oPRL treatment caused an increase in total TH protein levels at 1 h, 2 h, 4 h, and 72 h (Figure 7D).

## 4. Discussion

This study reports distinct time-dependent changes in PRLR activation of the JAK2-STAT5 signaling pathway in the MBH and in TH phosphorylation state, TH catalytic activity, and *Th* mRNA levels in TIDA neurons. Increased TH phosphorylation state and TH catalytic activity were closely correlated, but augmentation of *Th* mRNA levels occurred with a markedly delayed time course. In addition, this study reports the lack of a direct effect of the PRLR-STAT5 signaling pathway on *Th* promoter activity.

The best-defined intracellular signaling pathway for PRLR is the JAK2-STAT5 pathway, where phosphorylated STAT5 acts as a transcription factor binding GAS elements in PRL-responsive genes to elicit a biological response [23,24,25]. Most TH-expressing cells in hypothalamic cultures or TIDA neurons in the arcuate nucleus express primarily the long-form PRLR, supporting a possible direct effect of PRL on TIDA neurons [13,18,19,20]. As with other PRL-responsive tissues, this study confirms that the JAK2-STAT5 signaling pathway was rapidly activated and that STAT5B was the isoform activated by PRLR signaling in cells within the MBH of rats. These data are consistent with previous studies using rats or *Stat5b*-deficient mice, which show that the STAT5B isoform is activated and translocated to the nucleus in TIDA neurons [27,28,30]. In contrast to these previous studies specifically identifying phosphorylated STAT5 in TIDA neurons, western blot analysis permits a more accurate time course analysis, but with the caveat that other PRLR-expressing cells in the MBH may contribute to the phosphorylated JAK2 and STAT5 signals [13,19,20]. In addition, there are three neuroendocrine dopaminergic neuronal populations in the hypothalamus [2,5,17]. The MBH punch used for the current study contained the majority of the TIDA population but may have contained some tuberohypophyseal dopamine neurons, but did not contain periventricular hypophyseal dopamine neurons or zona incerta dopamine neurons. The PRL-induced increase in JAK2 phosphorylation was the initial rapid response at 15 min post-injection, followed by peak levels of STAT5 phosphorylation at 30–60 min JAK2-STAT5 pathway activation was transient, as phosphorylation levels for JAK2 returned to basal levels by 30 min, and STAT5 phosphorylation decreased by 2 h. In agreement with a previous report that PRL-induced activation specifically involves the STAT5 signaling pathway and does not involve STAT1 and STAT3 pathways [27], pilot studies in our lab using western blot analysis also showed no phosphorylation changes in STAT1 and STAT3 proteins within the timeframe of STAT5 activation after oPRL injection (Arbogast LA unpublished data). Although not explored in this study, intracellular negative signaling pathways involving the suppressors of cytokine signaling (SOCS) proteins and cytokine-inducible SH2 protein (CISH) likely play a role in the cessation of PRLR signaling, as evidenced in other tissues [25] and have been suggested for TIDA neurons [37,38].

We previously reported a modest increase in *Th* mRNA levels in the arcuate nucleus after 3 days of oPRL treatment in ovariectomized rats and a marked decrease after bromocriptine treatment, which was reversed by oPRL treatment [16]. Taken together with the confirmation in the present study that bromocriptine treatment reduced *Th* mRNA levels, these data indicated that low basal circulating PRL levels were sufficient to sustain *Th* mRNA levels in TIDA neurons and that elevated PRL for 3 days was able to further increase *Th* gene expression in TIDA neurons, both modestly without and markedly with bromocriptine treatment [16]. These data led us to hypothesize that PRL may be acting by the canonical PRLR pathway via direct STAT5 binding to one or more of the three putative GAS elements in the *Th* promoter. The *Th* promoter contains three putative GAS sites (TTCNNNGAA) at −1061 bp, −2757 bp and −5121 bp. The GAS element at −1061 bp (TTCCTCGAA) was considered the most likely target for pSTAT5B binding to mediate the PRL-induced increase in *Th* gene expression. However, *Th* promoter activity was unchanged in constructs containing one or more of the putative GAS elements, in spite of STAT5B being phosphorylated and translocated to the nucleus in response to PRL stimulation of CAD cells. Similarly, EMSA analysis did not reveal constitutively active STAT5B binding to the putative GAS sequence at −1061 bp. It is noteworthy that when the internal nucleotides (TTCNNNGAA) were mutated to the nucleotides in the GAS element of the casein promoter, this DNA sequence bound constitutively active STAT5B, suggesting that these internal variable nucleotides play an important function in STAT5 binding. This finding is consistent with similar core (TTC(T/C)N(G/A)GAA) for the GAS element reported previously for STAT5A and STAT5B [39]. Taken together, our data do not support that the change in *Th* gene expression was mediated directly by PRLR signaling through the JAK2-STAT5B pathway, with STAT5B acting as a transcription factor on the TH promoter.

Given that PRL did not directly increase *Th* promoter activity, the next experiments were designed to define the time required to observe the increase in *Th* gene expression. For these experiments, we used a model of continuous oPRL systemic infusion into the jugular vein. This method of administration resulted in elevated levels of PRL but within the physiological range. Consistent with the lack of PRLR signaling on *Th* promoter activity and the lack of direct binding of STAT5B to the *Th* promoter, *Th* mRNA signal levels were not increased within 24 h of oPRL infusion in the presence or absence of bromocriptine treatment. In our previous studies [16], oPRL administration elicited a modest increase in *Th* gene expression after 3 days, whereas in the current study, 3 days of oPRL infusion did not result in a change in *Th* mRNA levels. This difference may be due to the discontinuous versus continuous mode of oPRL administration in the previous and current study, respectively, or to the magnitude of the PRL elevation. In the previous study [16], oPRL was administered at 4 mg/kg s.c. at 8 h intervals, which results in very high oPRL levels after each injection and then a decline before the next injection. In this same study, haloperidol administration using a pellet for continuous release of approximately 200 ng/mL endogenous PRL did not alter *Th* gene expression after 3 days [16]. The haloperidol-induced PRL elevation closely resembled the constant infusion method of PRL, resulting in 200–500 ng/mL in the present study, supporting that continuously elevated PRL at moderately high levels is not sufficient to change *Th* gene expression at 3 days. However, in both the current and previous study [16], *Th* gene expression was elevated by 7 days of continuous administration of oPRL and rPRL using an osmotic minipump, resulting in PRL levels of approximately 100 ng/mL. Taken together, these data suggest that the timing, magnitude and mode of elevating PRL levels play a role in the PRL-induced elevation of *Th* gene expression in TIDA neurons. The PRL-induced increase in *Th* gene expression does not happen within a time frame consistent with direct STAT5B transactivation of the *Th* gene, but likely may be due to chronic alterations in TIDA neurons that require days.

Another important regulatory mechanism for TH activity is post-translational phosphorylation of the TH protein. The N-terminal domain of TH contains four serines (Ser8, Ser19, Ser31 and Ser40), which serve as regulatory phosphorylation sites [7,8,9]. Analysis of TH catalytic activity by DOPA accumulation in the ME reflects the influences of both TH phosphorylation state and expression of TH protein in TIDA neurons. It is notable that in the present study, the increase in TH enzyme activity closely corresponded to the increase in Ser40 phosphorylation state of TH, which was evident by 2 h after the initiation of oPRL infusion. This increase in TH Ser40 phosphorylation and TH activity in the ME was sustained for 72 h. Ser40 can be phosphorylated by a range of protein kinases, most notably the cyclic nucleotide-dependent protein kinases A and G and protein kinase C [7,8,9]. We previously observed that H-8, a cyclic nucleotide-dependent protein kinase inhibitor, reversed the PRL-induced increase in TH activity in the ME from adult ovariectomized rats [12]. Subsequently, Ma et al. [13] reported that H-89, a more specific protein kinase A inhibitor, blocked the PRL-induced increase in catecholamine synthesis in hypothalamic cell cultures. However, H-8 and H-89 may inhibit other kinases as well, albeit at higher IC50s [40,41]. Although these pharmacological data support a role for a cyclic nucleotide-dependent protein kinase, there is currently little evidence that PRL signaling through the JAK2-STAT5B pathway directly activates protein kinase A or protein kinase G. Ser40 can also be rapidly phosphorylated by protein kinase C, which has been implicated in the short-term activation of TH in TIDA neurons using hypothalamic slices in vitro, an effect which was inhibited by a potent and selective inhibitor of protein kinase C [10]. Moreover, bisindolymaleimide I, a protein kinase C inhibitor, prevented the PRL-induced increase in catecholamine synthesis in hypothalamic cell culture [13]. The exact pathway(s) targeted by these inhibitors to suppress or reverse the PRL-induced increase in dopamine synthesis require further investigation.

Although less consistent in timing and with less correlation to TH enzyme activity, oPRL also increased the TH phosphorylation state of Ser31 and Ser19 in the ME. PRLR signaling can activate the mitogen-activated protein kinase (MAPK) pathway, leading to activation of extracellular signal-regulated (ERK) [24,26]. Ser31 is a target of ERK 1/2, and its phosphorylation state was sporadically elevated during oPRL treatment in the current study. These data suggest that ERK 1/2 activation and TH Ser31 phosphorylation are unlikely to be the driving mechanism for increased TH activity, but may contribute to TH activation. Ma et al. [13] reported that PRL increased ERK 1/2 phosphorylation in hypothalamic cell cultures, and this effect was blocked by both PD98059, a mitogen-activated protein kinase kinase 1 (MEK1) inhibitor, and bisindolymaleimide I, a protein kinase C inhibitor; furthermore, PD98059 decreased basal, but not PRL-induced, catecholamine synthesis. These authors concluded that ERK 1/2 phosphorylation was mediated by protein kinase C [13]. Ser19 is a target for calcium-calmodulin-dependent protein kinase II, and its phosphorylation state was modestly elevated at 2 h and 4 h after initiating oPRL infusion. Although not reaching statistical significance, there was a trend for elevated Ser19 phosphorylation between 30 min and 24 h. In our previous study [12], TH enzyme activity in the ME was inhibited by W-7, a calmodulin antagonist, and partially reversed by KN62, a calcium/calmodulin-dependent protein kinase II inhibitor. However, KN93, a selective calcium/calmodulin-dependent protein kinase II inhibitor, did not reverse the PRL-induced increase in TH activity in the ME, whereas KN92, the inactive analog, showed some inhibitory effect [12]. Similar effects of KN93 and KN92 on PRL-induced catecholamine synthesis were observed in hypothalamic cell cultures [13]. Taken together, these data from previous studies support calcium and calmodulin involvement in the PRL-induced activation of TH, but do not support that calcium-calmodulin-dependent kinase II was the effector. Although the JAK2-STAT5 signaling pathway is the major PRLR signaling pathway and is activated in TIDA neurons, other intracellular signaling pathways may be activated by PRL binding to PRLR, but their activation and role in serine phosphorylation events elicited by PRL feedback still require further investigation.

The significant decrease in endogenous rPRL was observed as early as 30 min after the initiation of oPRL infusion. The suppression of endogenous PRL release occurred at the same time as the first significant increase in TH Ser40 phosphorylation, before a significant change in TH activity in the ME at 2 h and a long time prior to the change in *Th* mRNA levels in the arcuate nucleus at 7 days. Some PRL effects in TIDA neurons may stem from rapid changes in the electrical activity of TIDA neurons or changes in intracellular calcium. With the administration of PRL, TIDA neurons depolarize and switch to an increase in the firing rate within 5 min exhibiting tonic action potential discharge [42,43,44]. Indeed, using mouse brain slices, PRL elicits a reversible increase in firing rate of the majority of TIDA neurons independently of sex or reproductive state and an increase in dopamine release in the ME of virgin male and female rats [44]. The firing pattern in TIDA neurons closely correlates with the dynamics of dopamine release at both the terminal and somatodendritic levels [45]. The relatively rapid decrease in circulating PRL levels in the present study may likely be attributed to an increase in dopamine secretion at the level of the ME before other mechanisms follow. PRL causes a rapid increase in intracellular calcium in arcuate nucleus neurons [46]. Taken together, these data support a change in firing rate in TIDA neurons, contributions of calcium-dependent mechanisms, and dynamic patterns of dopamine secretion likely contribute to early events in TIDA neurons in response to increased PRL and mediate the decrease in endogenous PRL secretion.

Ovariectomized female rats were used for the study to examine the effects of PRL in the absence of ovarian steroid input. The ovariectomized rat model does not have the complexities of intact rats, in which estradiol and progesterone show cyclic changes during the reproductive cycle and distinctive profiles in pregnancy and lactation. Estradiol and progesterone can alter TIDA neuronal activity and play a role in TIDA neuronal activity during the reproductive cycle, pregnancy and lactation [32,47,48,49,50,51,52]. Estradiol has both stimulatory and inhibitory actions on TIDA neurons, with early stimulatory effects being mediated by the estradiol-induced increase in PRL secretion [49,53]. Taken together, these previous studies indicate complex interactions of steroid and lactogenic hormones in TIDA neurons of intact rats. The effects of PRL in the presence of steroid hormones and during different endocrine states need further exploration.

## 5. Conclusions

Taken together with previous studies, these data support the notion that continuous PRL elevation for a period of time initiates an integrated cascade of intracellular mechanisms in TIDA neurons. An early event was suppression of endogenous PRL release within 30 min, which persisted for at least 72 h in the present study. Electrophysiological changes and increased intracellular calcium in TIDA neurons likely contributed to an increase in dopamine secretion, leading to this early suppression of PRL secretion. Dopamine synthesis was augmented by serine phosphorylation in the regulatory domain of TH, particularly Ser40, concomitant with an increase in TH catalytic activity in the ME by 2 h, which endured for at least 72 h. Pathway inhibitors have implicated cyclic nucleotide-dependent protein kinases, protein kinase C, and calcium-dependent pathways in these PRL-induced TH phosphorylation changes, but the exact pathways have not been unequivocally identified. The JAK2-STAT5B pathway was rapidly and transiently activated in the MBH 15 to 60 min after PRL administration. Although essential for the increase in TIDA neuronal activity, the connections between PRLR-JAK2-STAT5 and membrane receptor-regulated protein kinases involved in serine phosphorylation of TH have not been clearly defined. *Th* gene expression was increased after prolonged PRL treatment of 7 days in the present study and 3 days in a previous study [16]. However, PRL did not appear to act via the conventional PRLR-JAK2-STAT5 pathway, involving direct STAT5 transactivation of the *Th* gene. *Th* promoter activity was not increased by PRL treatment, and STAT5B did not bind putative GAS elements in the *Th* promoter. Low basal circulating PRL levels have a role in maintaining *Th* gene expression, as evidenced by the decrease in TH gene expression after bromocriptine treatment. With higher PRL levels, the mode of administration and the absolute magnitude of the PRL levels appear to play a role in the timing of the PRL-induced increase in *Th* gene expression. One might postulate that the changes in *Th* gene expression are dependent on sustained activation of TIDA neurons, but the mechanisms still need to be explored.

## Data Availability

The original contributions presented in this study are included in the article/Supplementary Materials. Further inquiries can be directed to the corresponding author.

## Supplementary Materials

The following supporting information can be downloaded at: https://www.mdpi.com/article/10.3390/cells14090642/s1. Supplemental Figure S1 shows *Th* promoter-luciferase reporter data in PC-12 and N-39 cells. Supplemental Figures S2–S6 show representative blots for Figure 2 and Figure 7.

## Abbreviations

Adenosine triphosphate (ATP); Base pair (bp); Bovine serum albumin (BSA); Bromocriptine (BROMO); Complementary DNA (cDNA); Complementary RNA (cRNA); Cytomegalovirus (CMV); Deoxyribonucleic acid (DNA); Dihydroxyphenylalanine (DOPA); Disintegrations per minute (dpm); Dithiothreitol (DTT); Double-stranded DNA (dsDNA); Electrophoretic Mobility Shift Assay (EMSA); Ethylenediaminetetraacetic acid (EDTA); Extracellular signal-regulated kinase (ERK); Fetal bovine serum (FBS); Gamma interferon activation site (GAS); Guanosine triphosphate (GTP); High performance liquid chromatography (HPLC); Horseradish peroxidase (HRP); Insulin-Transferrin; Selenium (ITS); Intravenously (i.v.); Janus Kinase 2 (JAK2); Median Eminence (ME); Mediobasal hypothalamus (MBH); Messenger ribonucleic acid (mRNA); Mitogen-activated protein kinase (MAPK); Mitogen-activated protein kinase kinase 1 (MEK1); Nuclear receptor-related 1 (NURR1); Ovine prolactin (oPRL); Phospho-Janus kinase 2 (pJAK2); Phospho-signal activator and transducer of transcription 5 (pSTAT5); Phospho-tyrosine hydroxylase (pTH); Polymerase chain reaction (PCR);Polyvinylidene fluoride (PVDF); Prolactin (PRL); Prolactin receptor (PRLR); Quantitative reverse transcriptase polymerase chain reaction (qRT-PCR); Rat prolactin (rPRL); Serine (Ser); Signal activator and transducer of transcription (STAT); Signal activator and transducer of transcription 5 (STAT5); Sodium dodecyl sulfate-polyacrylamide gel electrophoresis (SDS-PAGE); Subcutaneously (s.c.); Tris-buffered saline (TBS); Tuberoinfundibular dopaminergic (TIDA); Tyrosine Hydroxylase (TH).
