# Supplementary material for: Prolactin Mediates Distinct Time Course Regulation of Tyrosine Hydroxylase Phosphorylation and Gene Expression in Tuberoinfundibular Dopaminergic Neurons of Female Rats
Source: Cells. 2025 Apr 27;14(9):642. doi: 10.3390/cells14090642 (PMC12071785; doi:10.3390/cells14090642)
Supplement: Supplementary file 1 [file cells-14-00642-s001.zip › cells-3415304-supplementary.pdf]

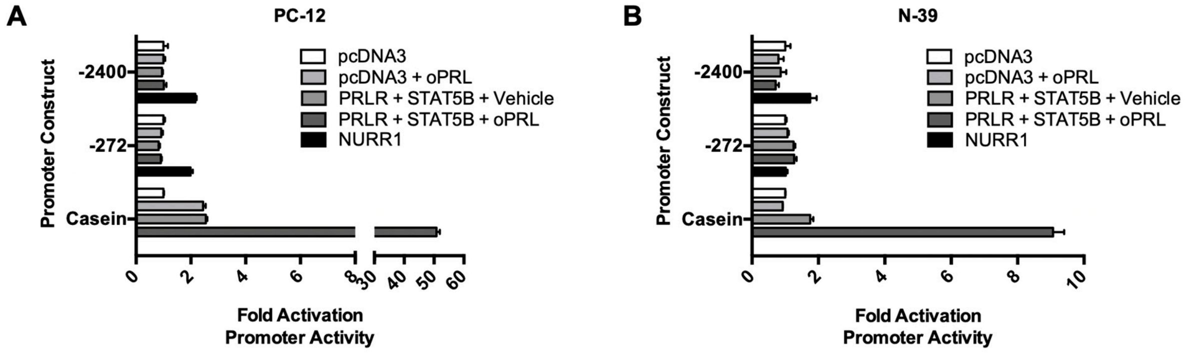

Figure S1. Effects of PRL-STAT5B signaling on rat *Th* promoter constructs. **(A)** PC-12 cells or **(B)** N-39 cells were co-transfected with the indicated *Th* promoter-luciferase or casein promoter-luciferase constructs as indicated and with pcDNA3, CMV-rPRLR, CMV-rSTAT5B, or rNURR1 constructs as indicated. Cells were also co-transfected with CMV-renilla luciferase to determine transfection efficiency. Cells were treated with vehicle or oPRL (1000 ng/mL) for 24 h, followed by dual luciferase assays. Luciferase values were normalized to the pcDNA3+ vehicle values to determine fold activation. A −0.5 kb casein promoter fragment containing a GAS element was used as a control for PRL-driven promoter activation. Each value is a mean ±SE of 3 independent luciferase assays.

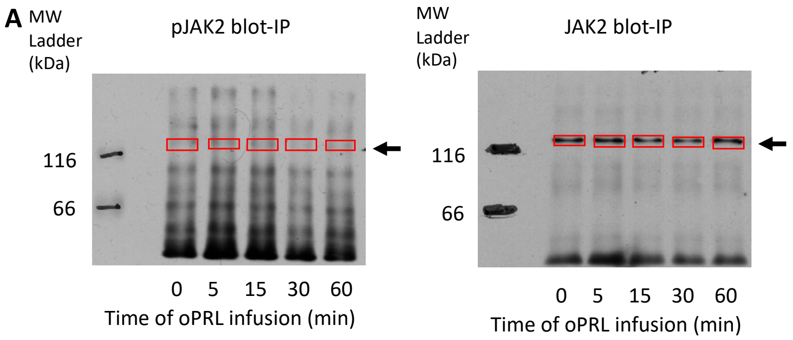

Density Values (volume)

Time (min)	pJak2	JAK2
0	159	1505
5	333	1650
15	289	1036
30	157	857
60	140	1502

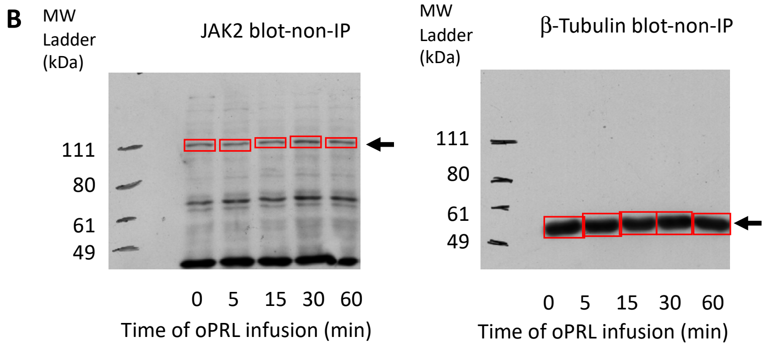

Density Values (volume)

Time (min)	β-Tubulin	Jak2
0	3878	256
5	3828	269
15	3609	301
30	3995	378
60	3818	316

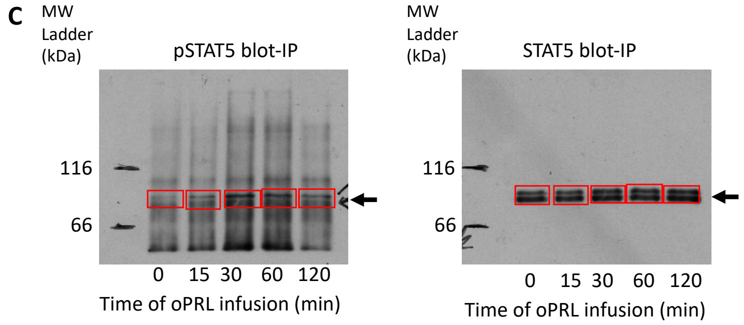

Density Values (volume)

Time (min)	pSTAT5	STAT5
0	1001	2417
15	1403	2709
30	2506	2801
60	2310	2594
120	1139	2494

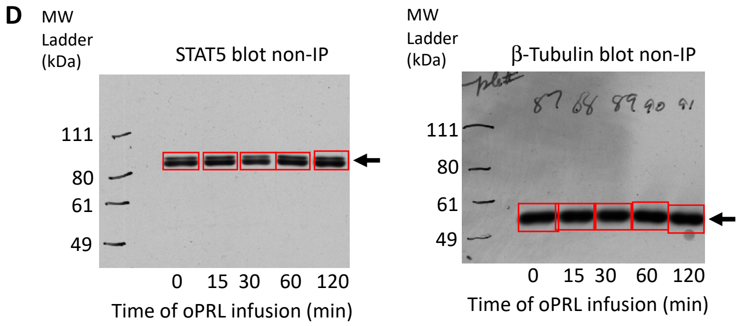

Density Values (volume)

Time (min)	STAT5	β-Tubulin
0	2403	4498
15	2585	4658
30	2182	4642
60	2465	4710
120	2360	4556

Figure S2. Representative blots for Figure 2 in manuscript. **(A,C)** MBH samples were immunoprecipitated and then subjected to western blot as described in materials and methods.

Blots for phospho-proteins are on left and total proteins are on the right. **(B,D)** MBH input non-immunoprecipitated (non-IP) samples. Blots for JAK2 and STAT5 proteins are on the left and β-tubulin are on the right. Molecular weight markers (kDa) are indicated to left of blots. Arrows indicate band of interest. Red boxes are representative of regions of interest analyzed for density. Density values are listed in tables. That ratios of pJAK2:JAK2 densities and pSTAT5 densities and %Control values were calculated for each blot. Data analysis was performed on the %Control values as described in materials and methods.

Representative pTH-Ser 40 Blots on Film

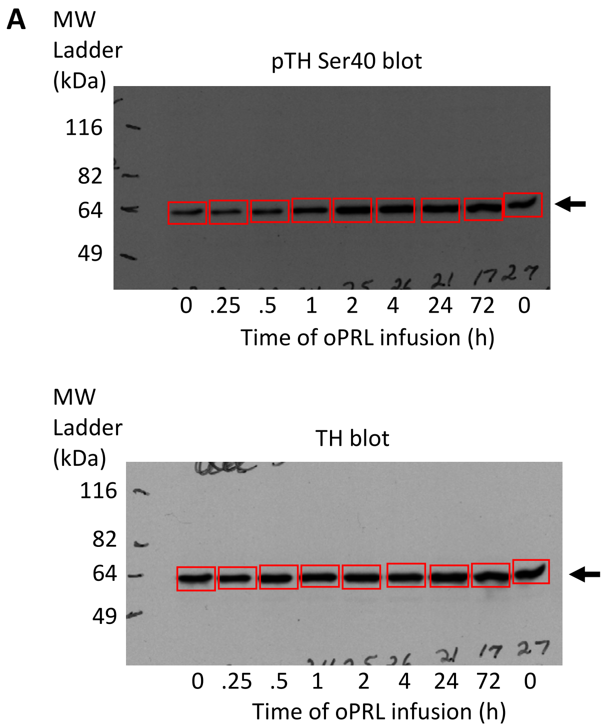

	Density Values (volume)	
Time (h)	pTH-Ser40	TH
0 (mean)	1309	4035
.25	716	3650
.5	939	4737
1	1642	4674
2	3381	4579
4	3364	3795
24	3011	4888
72	3164	4196

Representative pTH-Ser 40 Blots from Odyssey Analysis

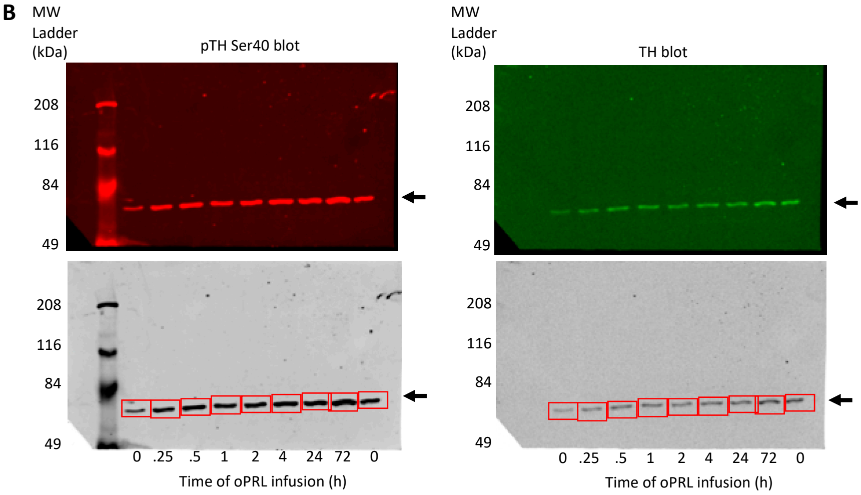

ID/Name	Integ.Intensity	Treatment	Band
1-700	2.56	Control	Ser 40
1-800	0.37	Control	TH
2-700	8.44	oPRL-15m	Ser 40
2-800	0.55	oPRL-15m	TH
3-700	14.14	oPRL-30m	Ser 40
3-800	0.83	oPRL-30m	TH
4-700	8.97	oPRL-1h	Ser 40
4-800	0.84	oPRL-1h	TH
5-700	10.75	oPRL-2h	Ser 40
5-800	0.73	oPRL-2h	TH
6-700	16.07	oPRL-4h	Ser 40
6-800	0.9	oPRL-4h	TH
7-700	15.79	oPRL-24h	Ser 40
7-800	0.96	oPRL-24h	TH
8-700	26.39	oPRL-72h	Ser 40
8-800	1.28	oPRL-72h	TH
9-700	9.01	Control	Ser 40
9-800	1.01	Control	TH

Figure S3. Representative blots for pTH Ser40 in Figure 7 of manuscript. Western blotting was performed as described in materials and methods and analyzed using film and Molecular Dynamics Densitometer **(A)** or using Licor Odyssey Imager **(B)**. Molecular weight markers (kDa) are indicated to left of blots. Arrows indicate band of interest. Red boxes are representative of regions of interest analyzed for density. Density values are listed in tables. For Odyssey Imager,

700nm (red) channel represents pTH-Ser40 and 800nm (green channel) represents TH. For Odyssey blots, 700nm and 800nm channels were analyzed simultaneously, but molecular weight markers were detected only in the 700nm channel. Note each blot contains 2 control (0 time) samples. That ratios of pTH-Ser40:TH densities and %Control values were calculated for each blot. Data analysis was performed on the %Control values as described in materials and methods.

Representative pTH-Ser 31 Blots on Film

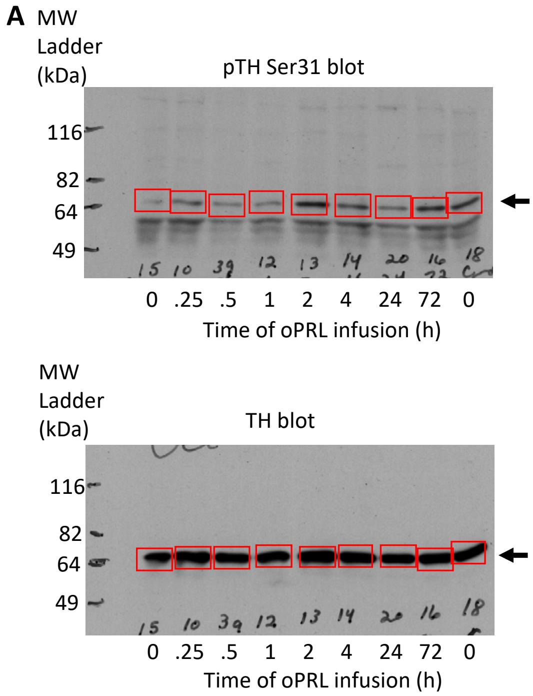

	Density Values (volume)	
Time (h)	pTH-Ser31	TH
0 (mean)	461	4064
.25	547	6151
.5	240	4145
1	256	5604
2	1256	7123
4	852	6341
24	431	5425
72	902	5167

Representative pTH-Ser 31 Blots from Odyssey Analysis

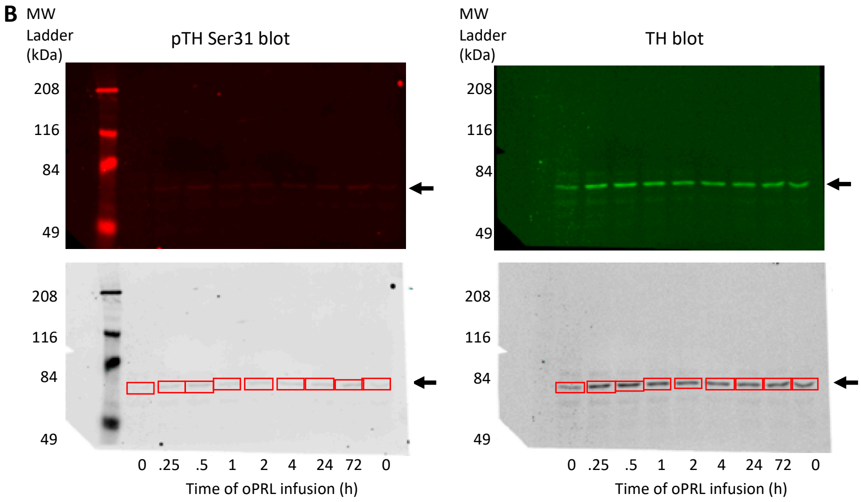

ID/Name	Integ.Intensity	Treatment	Band
1-700	0.64	Control	Ser 31
1-800	1.12	Control	TH
2-700	0.94	oPRL-15m	Ser 31
2-800	1.8	oPRL-15m	TH
3-700	1.09	oPRL-30m	Ser 31
3-800	1.9	oPRL-30m	TH
4-700	0.65	oPRL-1h	Ser 31
4-800	1.63	oPRL-1h	TH
5-700	0.65	oPRL-2h	Ser 31
5-800	1.34	oPRL-2h	TH
6-700	0.44	oPRL-4h	Ser 31
6-800	1.33	oPRL-4h	TH
7-700	0.78	oPRL-24h	Ser 31
7-800	1.48	oPRL-24h	TH
8-700	1.14	oPRL-72h	Ser 31
8-800	1.55	oPRL-72h	TH
9-700	0.49	Control	Ser 31
9-800	1.28	Control	TH

Figure S4. Representative blots for pTH Ser31 in Figure 7 of manuscript. Western blotting was performed as described in materials and methods and analyzed using film and Molecular Dynamics Densitometer **(A)** or using Licor Odyssey Imager **(B)**. Molecular weight markers (kDa) are indicated to left of blots. Arrows indicate band of interest. Red boxes are representative of regions of interest analyzed for density. Density values are listed in tables. For Odyssey Imager, 700nm (red) channel represents pTH-Ser31 and 800nm (green channel) represents TH. For

Odyssey blots, 700nm and 800nm channels were analyzed simultaneously, but molecular weight markers were detected only in the 700nm channel. Note each blot contains 2 control (0 time) samples. That ratios of pTH-Ser31:TH densities and %Control values were calculated for each blot. Data analysis was performed on the %Control values as described in materials and methods.

Representative pTH-Ser 19 Blots on Film

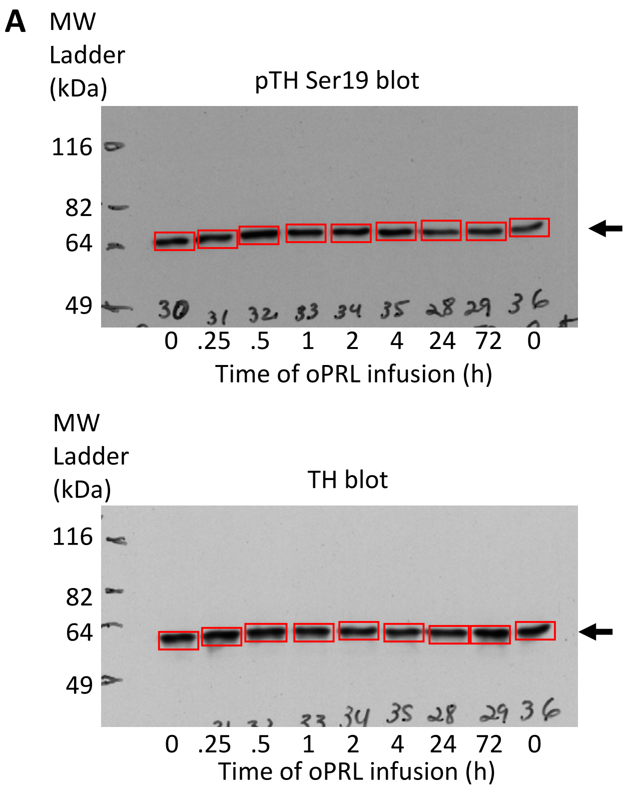

Density Values (volume)

Time (h)	pTH-Ser19	TH
0 (mean)	1722	2946
.25	1910	3367
.5	3290	3170
1	2211	2798
2	2562	2687
4	2259	2086
24	1312	2372
72	1291	3562

Representative pTH-Ser 19 Blots from Odyssey Analysis

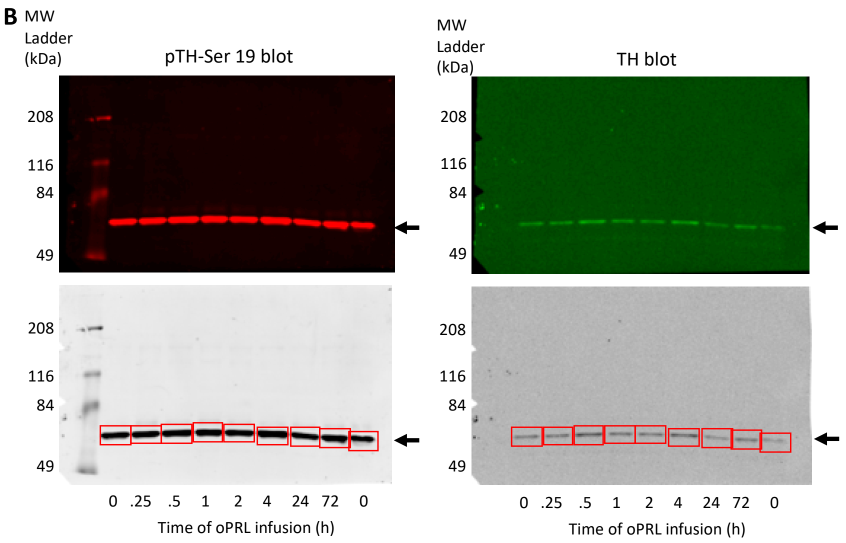

ID/Name	Integ.Intensity	Treatment	Band
1-700	65.22	Control	Ser 19
1-800	0.57	Control	TH
2-700	87.13	oPRL-15m	Ser 19
2-800	0.55	oPRL-15m	TH
3-700	111.43	oPRL-30m	Ser 19
3-800	1	oPRL-30m	TH
4-700	93.67	oPRL-1h	Ser 19
4-800	0.62	oPRL-1h	TH
5-700	80.56	oPRL-2h	Ser 19
5-800	0.57	oPRL-2h	TH
6-700	118.59	oPRL-4h	Ser 19
6-800	0.86	oPRL-4h	TH
7-700	58.49	oPRL-24h	Ser 19
7-800	0.41	oPRL-24h	TH
8-700	72.72	oPRL-72h	Ser 19
8-800	0.62	oPRL-72h	TH
9-700	38.82	Control	Ser 19
9-800	0.32	Control	TH

Figure S5. Representative blots for pTH Ser19 in Figure 7 of manuscript. Western blotting was performed as described in materials and methods and analyzed using film and Molecular Dynamics Densitometer **(A)** or using Licor Odyssey Imager **(B)**. Molecular weight markers (kDa) are indicated to left of blots. Arrows indicate band of interest. Red boxes are representative of

regions of interest analyzed for density. Density values are listed in tables. For Odyssey Imager, 700nm (red) channel represents pTH-Ser19 and 800nm (green channel) represents TH. For Odyssey blots, 700nm and 800nm channels were analyzed simultaneously, but molecular weight markers were detected only in the 700nm channel. Note each blot contains 2 control (0 time) samples. That ratios of pTH-Ser19:TH densities and %Control values were calculated for each blot. Data analysis was performed on the %Control values as described in materials and methods.

Representative TH Blots on Film

A

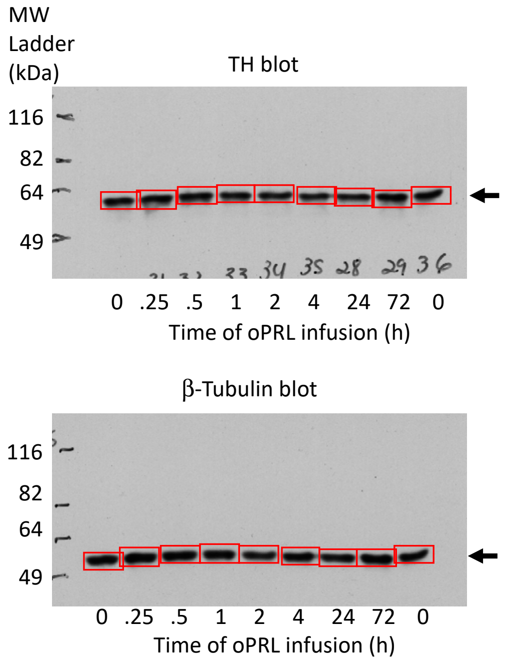

Density Values (volume)

Time (h)	TH	β -Tubulin
0 (mean)	4759	4241
.25	5517	4290
.5	5821	4453
1	5418	3889
2	5299	3478
4	5103	3416
24	5382	4408
72	5483	3708

B

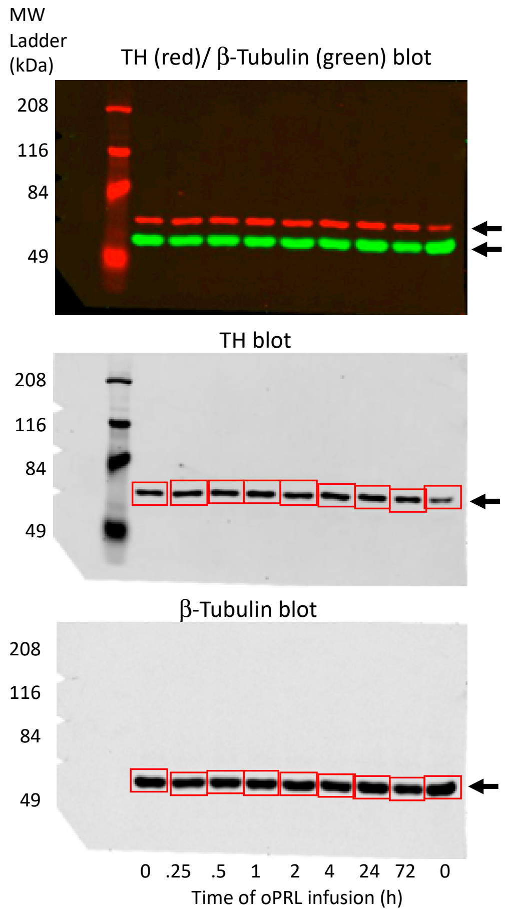

ID/Name	Int. Intensity	Treatment	Band
1-700	9.73	Control	TH
2-700	13.04	oPRL-15m	TH
3-700	12.87	oPRL-30m	TH
4-700	17.06	oPRL-1h	TH
5-700	13.16	oPRL-2h	TH
6-700	15.77	oPRL-4h	TH
7-700	15.33	oPRL-24h	TH
8-700	10.58	oPRL-72h	TH
9-700	4.95	Control	TH
10-800	20.34	Control	bTubulin
11-800	17.04	oPRL-15m	bTubulin
12-800	17.25	oPRL-30m	bTubulin
13-800	17.8	oPRL-1h	bTubulin
14-800	18.67	oPRL-2h	bTubulin
15-800	17.38	oPRL-4h	bTubulin
16-800	21.12	oPRL-24h	bTubulin
17-800	15.42	oPRL-72h	bTubulin
18-800	23.41	Control	bTubulin

Figure S6. Representative blots for total TH in Figure 7 of manuscript. Western blotting was performed as described in materials and methods and analyzed using film and Molecular Dynamics Densitometer **(A)** or using Licor Odyssey Imager **(B)**. Molecular weight markers (kDa) are indicated to left of blots. Arrows indicate band of interest. Red boxes are representative of regions of interest analyzed for density. Density values are listed in tables. For Odyssey Imager, 700nm (red) channel represents TH and 800nm (green channel) represents β-Tubulin. For Odyssey blots, 700nm and 800nm channels were analyzed simultaneously, but molecular weight markers were detected only in the 700nm channel. Note each blot contains 2 control (0 time) samples. That ratios of TH:β-Tubulin densities and %Control values were calculated for each blot. Data analysis was performed on the %Control values as described in materials and methods.
